# Treatment of Disease in Children

**Published:** 1890-09

**Authors:** 


*Treatment of Disease in Children.* By ANGEL MONEY, M.D. Second Edition. London: H. K. LEWIS. 1890.

Containing more than is implied in its main title, this book is a very useful one. Fair space is given to the outlines of diagnosis, which must be accurate if treatment is to be anything but guess-work. The aim of this book necessitates, of course, the suggestion of a large variety of drugs which the patient would often doubtless be better without. The child must be an exceptional one who would, without an injurious tussle, take some of the mixtures recommended by Dr. Money. But as with these suggestions the reader gets an extensive selection of drugs, the alternative modes of treatment referred to by the author are no drawback to the book, which contains much that is helpful in daily practice.

Dr. Money's descriptions are clear, and are nearly always given in good English; but when, in reference to the difficulty sometimes experienced in the administration of cod-liver oil, he says that some children frequently "raise" the oil after it has been taken, we seem to detect a trans-Atlantic phrasing that has not yet been accepted in these islands. We prefer the euphemism of an American lady, who said to a friend of ours upon hearing that he was always ill on board ship: "Does your nausea take action?"

